# Facilitators and barriers to help-seeking behavior for symptoms in patients with lung cancer in China—a qualitative study

**DOI:** 10.3389/fpsyg.2025.1718034

**Published:** 2026-01-12

**Authors:** Jingying Yang, Yingqi Yin, Guilin Yu, Feng Yu

**Affiliations:** 1 Department of Nursing, Medical College of Wuhan University of Science and Technology, Wuhan, Hubei, China; 2 Department of Cardiovascular, Wuhan Asia General Hospital, Wuhan, Hubei, China; 3 Tumor Center of Union Hospital Affiliated to Tongji Medical College of Huazhong University of Science and Technology, Wuhan, Hubei, China

**Keywords:** health belief model, help-seeking behavior, influencing factors, lung cancer, qualitative study

## Abstract

**Objective:**

Guided by the Health Belief Model (HBM), this study explores facilitators and barriers influencing help-seeking behavior for symptoms among Chinese patients with lung cancer, aiming to inform strategies for earlier diagnosis and improved patient outcomes.

**Methods:**

In-depth, face-to-face personal interviews were conducted with 14 lung cancer patients from a tertiary A-level hospital in Hubei province. The collected data were analyzed using thematic analysis.

**Results:**

Two main themes emerged—facilitators and barriers—along with six sub-themes: perceived threats, perceived benefits, self-efficacy, cues to action, perceived internal barriers, and perceived external barriers. Participants generally sought medical help when symptoms became sudden, severe, or significantly disrupted daily life. Key barriers included lack of health awareness, psychological acceptance barriers, and misdiagnosis.

**Conclusion:**

Relevant authorities should gradually improve public health awareness. Communities and the government should continuously enhance patients' healthcare experiences. At the same time, communities and families should provide patients with practical and emotional support to encourage timely medical help and promote healthier lifestyles.

## Introduction

With approximately 2.5 million new cases and over 1.8 million deaths worldwide, lung cancer is the leading cause of cancer morbidity and mortality in 2022 (Bray et al., 2024). lung cancer is the most common cancer type in China, with 1.0606 million new cases reported, with 733,300 resulting in death in 2022 (Han et al., 2024). The high mortality rate of lung cancer may be attributed to delays in seeking medical attention and late-stage diagnosis (Moffat et al., 2015; Hanna et al., 2020). Delays in seeking medical care may result in patients being diagnosed at more advanced stages of cancer (Lyratzopoulos et al., 2015). Patients diagnosed at later stages have lower cure rates and higher mortality risks (Tsai et al., 2020). Therefore, early help-seeking behavior and diagnosis are crucial for improving treatment outcomes and reducing disease burden (Dobson et al., 2014).

“Help-seeking behavior for cancer symptoms” refers to the process of seeking medical assistance and utilizing healthcare services upon detecting potential cancer symptoms (Momeni and Rafii, 2020). Previous studies explored the factors that influence help-seeking behavior in cancer patients. First, the recent Women, power and cancer Lancet Commission also highlighted a myriad of factors and overlapping forms of discrimination (e.g., age, ethnicity, socioeconomic status, gender identity) that are likely to challenge access to early detection and diagnosis of cancer for women (Ginsburg et al., 2023). Second, limited awareness of cancer symptoms and difficulty recognizing possible warning signs are common reasons why patients delay seeking help (Kailemia et al., 2020; McCutchan et al., 2021). Third, sociocultural factors such as personal beliefs and traditional practices also influence whether patients seek medical care (Akuoko et al., 2017). Fear of a cancer diagnosis can either prompt or delay help-seeking, depending on its intensity, duration, context, coping skills, and sociodemographic factors (Niksic et al., 2016). Finally, practical barriers such as time constraints and difficulty reaching healthcare facilities also delay care-seeking (Simon et al., 2010). Waiting more than 90 days to see a doctor greatly raises the risk of late-stage cancer diagnosis (Moodley et al., 2018).

The timeliness of seeking help for lung cancer symptoms is influenced by multiple factors. Firstly, because early-stage lung cancer is often asymptomatic or presents with non-specific symptoms, diagnosis can be significantly delayed (Birring and Peake, 2005; Koo et al., 2018). Additionally, some lung cancer patients may mistakenly attribute their symptoms to pre-existing pulmonary or cardiac conditions, such as chronic obstructive pulmonary disease (COPD) or asthma, thereby remaining unaware of the underlying lung cancer (Cunningham et al., 2019; Friedemann Smith et al., 2016; Otty et al., 2023; Corner et al., 2006; Smith et al., 2009). In addition, lung cancer patients who are unwilling to accept their condition may fail to recognize symptoms or refuse to acknowledge them, while those with anxiety disorders might disregard these symptoms (Kotecha et al., 2021). Stigma and fear are closely associated with delays in seeking medical treatment among smokers (Carter-Harris, 2015; Marlow et al., 2015; Murray et al., 2017; Quaife et al., 2015). Delays in seeking medical help negatively impact timely disease diagnosis, patient quality of life, and healthcare costs (Walter et al., 2015). Finally, barriers to seeking help for lung cancer symptoms associated with the healthcare system include distrust in the competence of local physicians, limited access to healthcare services, high costs of consultations, and prolonged waiting time (Saab et al., 2021a; Ali et al., 2024; Elshami et al., 2024). Additionally, poor relationships with doctors, negative past experiences, and concerns about wasting doctors' time can further delay patients from seeking medical assistance (Jones et al., 2022).

Lung cancer survival rates are generally lower than those of other cancers, such as pancreatic, prostate, colorectal, and breast cancer (He et al., 2016). Previous research on help-seeking behavior for cancer symptoms has focused mainly on a few types, such as breast, ovarian, and colorectal cancer, with far less attention given to lung cancer, especially in China (Yin et al., 2024; Harries et al., 2020; Mwaka et al., 2021; Ng et al., 2020; Blackmore et al., 2020; Reese et al., 2020; Oshiro and Kamizato, 2018). The Health Belief Model (HBM) is one of the most widely used theoretical frameworks in health behavior research to explain the intrapersonal decision-making process of health behavior (Janz and Becker, 1984). According to the HBM, people must be psychologically ready to change their health behaviors (Rosenstock et al., 1988). It mainly includes perceived susceptibility and severity of illness, perceived benefits, perceived barriers and self-efficacy. These factors are interconnected: individuals who perceive greater severity may be more motivated to act if they believe they can succeed (self-efficacy) and see the benefits outweighing barriers (Khani Jeihooni et al., 2021; Wondmu et al., 2022). Cues to action can further trigger behavior change (Tang et al., 2025).

This study aims to use HBM to investigate the factors influencing help-seeking behavior among Chinese patients with lung cancer, in order to provide support for developing optimized strategies for the early detection of lung cancer and improving patient outcomes.

## Methods

### Study design

This study employed a qualitative approach, guided by HBM, to explore factors influencing help-seeking behavior for symptoms among lung cancer patients. Semi-structured interviews were conducted with lung cancer patients at a tertiary Grade A hospital in Wuhan, Hubei Province, China. This study was reported in accordance with the COREQ checklist.

### Ethical considerations

Approval for this study was obtained through the Ethics Committee of the School of Medicine, Wuhan University of Science and Technology (Study ID: 2025001).

### Recruitment

We employed a purposive sampling method to recruit lung cancer patients from a tertiary Grade A hospital. The inclusion criteria were as follows: Meeting the diagnostic criteria for lung cancer (International Classification of Diseases, Tenth Revision, C34); Age ≥ 18 years; Patients being mentally clear and capable of articulating their thoughts; Voluntary participation with informed consent. The exclusion criteria included: Patients with communication barriers; Patients suffering from severe physical illnesses or significant mental disorders. Through iterative analysis, we found that the number of new codes dropped sharply after the 11th interview. No new codes emerged in the final two interviews, indicating that no additional issues were identified and the codebook had stabilized. The research team agreed that data saturation had been reached (Hennink et al., 2017). Ultimately, we identified 14 lung cancer patients as participants.

### Data collection

This study adopted a qualitative research method with one-to-one semi-structured interviews for collecting data. Data collection was carried out between January and February 2025. All members of our research team have taken qualitative research courses and have mastered interview techniques and data analysis methods. The interviews were conducted by a registered nurse with extensive clinical experience in oncology. Her professional background enabled her to better understand the illness experiences of lung cancer patients. The corresponding author, an accomplished researcher holding a PhD, provides strategic direction for the research. Prior to the interviews, we informed the participants of the main purpose and content of the study, and they signed the informed consent. The interview outlines include: “What were your initial symptoms?” “Why do you think these symptoms appeared? What were your feelings or thoughts? “What did you do after these symptoms appeared?” “Do you think you went to the hospital for a check-up in time? What were the reasons?” ? “What difficulties did you encounter while seeking help?” Interviews were carried out in quiet rooms. The interviewer simultaneously observes and records the participants' expressions, actions and other non-verbal behaviors. All voice recordings were converted into a total of 40,000 words, and the average interview duration was 25 min.

### Data collection

The data were analyzed using thematic analysis approach (Braun and Clarke, 2008). The first author (J. Yang) transcribed the interview recordings verbatim. The transcribed interviews were reviewed by a second researcher (Y.Yin) to ensure transcriptions were accurate, complete, and unbiased.

The thematic analysis followed the six phases described by Braun and Clarke (2008): (1) familiarizing ourselves with the data, (2) generation of codes, (3) searching for themes, (4) reviewing themes, (5) defining and naming themes, and (6) producing the report. First, two researchers (J. Yang and Y. Yin) repeatedly listened to the audio recordings and read the full transcripts to develop initial analytical insights. In the second phase, the first and second authors independently coded the data by using NVivo 12 Plus software, generating an initial code list. In the third phase, they developed a visual chart to group similar codes and integrate them into themes. All authors reviewed these themes and provided feedback. In the fourth phase, the first and second authors refined the thematic structure by discarding themes with insufficient supporting data or substantial overlap, returning to the original transcripts for verification. In the fifth phase, they finalized the definitions and names of the themes. The final phase involved all authors collaboratively drafting and finalizing the research report. The encoding results are presented in Appendix 1.

### Trustworthiness of qualitative data

To ensure rigor and trustworthiness, dependability, credibility, transferability, and confirmability of the data were evaluated (Thomas and Magilvy, 2011). First, any disagreement pertaining to the design, methods, data analysis, and results were all discussed in the research group until a consensus was reached. Second, two researchers independently coded all interview transcripts and analyzed them repeatedly. Discrepancies in coding or themes development were discussed with the corresponding author (G. Yu) to reach resolution. The final themes were reviewed and agreed upon by all authors to enhance consistency and minimize bias. Third, external researchers reviewed the coding process, theme generation and analytical logic to strengthen reflexivity and transparency. Additionally, three participants were invited to review the themes and supporting quotes to confirm that the codes and themes accurately reflected their feelings and experiences. Detailed records were kept throughout data collection, analysis, coding, and themes generation, and all steps were subject to supervision and team verification. Moreover, regarding the transferability of the study, the researcher described the research methods in detail to provide a reference for future research.

## Results

A total of 14 lung cancer patients, respectively, seven females and seven males, consented to be contacted for interviews, and their average age was 51. Table 1 provides an overview of the participants' general information. Based on the HBM, we identified two themes and six sub-themes (Table 2).

**Table 1:** General information of interview subjects.

**Participant**	**Gender**	**Age (years)**	**Education background**	**Marital status**	**Living area (rural/urban)**	**Stage of disease**
N1	Female	30	Junior college	Unmarried	Rural	IV
N2	Male	41	Middle school	Married	Rural	IV
N3	Male	60	Middle school	Married	Rural	IIIa
N4	Male	68	Primary school	Married	Rural	IV
N5	Female	62	Vocational school	Married	Urban	IV
N6	Female	35	Junior college	Married	Urban	IV
N7	Female	45	Middle school	Married	Urban	IV
N8	Female	44	Junior college	Married	Urban	IV
N9	Female	58	Primary school	Married	Rural	IV
N10	Male	45	Middle school	Married	Rural	IIIb
N11	Male	66	Middle school	Married	Urban	IIIa
N12	Male	61	Middle school	Married	Rural	IIIb
N13	Male	60	Illiteracy	Married	Rural	IIIb
N14	Female	39	Vocational school	Married	Rural	IV

**Table 2:** Themes and sub-themes.

**Themes**	**Sub-themes**	**Categories**
Facilitators	Perceived threats	Uncertainty about the illness Fear of sudden acute symptoms Interference with daily life
	Perceived benefits	Alleviation of stress Restoration of physical function Ability to earn an income
	Self-efficacy	Strong emotion regulation skills Confidence in rehabilitation capabilities
	Cues to action	Health insurance policy support Medical technical support Health information support Family support for medical visits Friend support for medical visits Mutual support among patients
Barriers	Perceived internal barriers	Lack of health awareness Conflict of personal responsibilities Psychological acceptance barriers Difficulties enduring treatment side effects Significant economic pressure
	Perceived external barriers	Misdiagnosis Long distance to medical facilities

### Theme 1: facilitators

#### Sub-theme 1: perceived threats

##### Uncertainty about the illness

Participants often felt confused and overwhelmed by persistent symptoms with no clear cause, which prompted them to seek medical evaluation to identify the underlying issue.

*Coughing usually gets better in about two weeks, but I'd been coughing for three or four weeks. I felt really lost, so I went to the hospital for a check-up. (N6)*

*I didn't understand why my body kept hurting—I was puzzled. I quickly went to the hospital near my home for an examination. (N1)*

*I kept coughing, and cold medicine didn't help at all. I felt completely confused and decided to go to the hospital for a check-up. (N9)*

##### Fear of sudden acute symptoms

Participants reported sudden acute symptoms in daily life, such as muscle spasms, severe pain, or coughing up blood. These symptoms frightened them and made them realize their health was seriously at risk.

*I suddenly had a severe muscle spasm at home and passed out from the pain. It really scared my husband, so we went straight to the emergency department at Zunyi Medical College for tests, including blood work and an MRI. (N14)*

*I noticed blood streaks in my cough, and I was really frightened—I knew something was wrong. I barely slept all night. Eventually, I went to the hospital for a check-up. (N6)*

##### Interference with daily life

Participants reported that persistent and unbearable symptoms disrupted their daily lives and caused significant distress. For example, chest and back pain interfered with sleep, and severe coughing made even drinking water difficult.

*After falling asleep, my leg pain eased, but I still had sharp pain in my chest and back. It's really affecting my daily life. (N1)*

*During everyday activities like climbing stairs or walking quickly, I get short of breath much more easily than before. I felt it was time to go to the hospital. (N2)*

*Even lifting things makes me more tired than usual. When I drink water, I start coughing nonstop and my voice gets hoarse. It's seriously disrupting my daily life. (N1)*

#### Sub-theme 2: perceived benefits

##### Alleviation of stress

Participants said that actively seeking help effectively reduced stress and eased anxiety. Consulting healthcare professionals gave them clear, expert answers, which lessened the confusion caused by uncertainty about their condition.

*Because we didn't know what was causing my cough, my family and I felt it would be reassuring to get it checked. After the examination, my stress really eased. (N1)*

*After I started coughing up blood, I felt under a lot of mental pressure. I believed seeing a doctor would help relieve that stress. (N6)*

*I think delaying medical care only adds to my stress. Avoiding tests won't make the problem go away. Staying emotionally stable will also help me with treatment later on. (N9)*

##### Restoration of physical function

Participants reported improvements in physical functioning after seeking help and receiving treatment. For example, they found climbing stairs less tiring and experienced fewer episodes of hoarseness.

*Since I got sick, I clearly noticed my body wasn't as strong as before. But after getting help and starting treatment, my stamina improved. (N6)*

*The doctor said recovery would take a long time, but not long after surgery, I was already able to move around on my own. (N14)*

*Thanks to treatment, my voice doesn't go hoarse as easily now. I truly hope I can one day drink a glass of water again as naturally and peacefully as I used to. (N1)*

##### Ability to earn an income

After initial treatment, participants felt that seeking help enabled them to regain their health, return to work, and better support their families. The ability to earn an income reinforced their perception of the benefits of timely medical care.

*Before treatment, my hands weren't as nimble. Now I can make handmade insoles at home and earn some money. (N14)*

*I really need to earn money to support my family. I hope to get back to work as soon as I recover. (N10)*

#### Sub-theme 3: self-efficacy

##### Strong emotion regulation skills

When facing health issues, the participants were able to regulate their emotions to maintain a stable mindset in dealing with the illness. Possessing good emotional regulation skills empowered them to seek medical help more proactively and comply with diagnostic and treatment arrangements.

*I usually maintain a calm attitude and don't burden myself with unnecessary thoughts. After coming to the hospital, I simply follow the doctor's guidance for treatment. (N5)*

*I feel I'm still young, so there's no need to worry excessively. I'll adhere to whatever treatment plan the doctor recommends. (N10)*

*I believe I have a positive mindset and can effectively regulate my emotions. I want to set a brave example for my daughter. Sometimes my younger sister cries because of my illness, and I even comfort her. (N14)*

##### Confidence in rehabilitation capabilities

Participants who firmly believed in their body's recovery ability were more proactive in following medical advice to complete treatments (e.g., medication, surgery, rehabilitation). This confidence drove them to attempt independent daily activities and keep seeking medical support.

*I am certain I can recover, and I am also willing to cooperate with treatment and exercise. (N14)*

*Although I cannot move as freely as before, I believe my body has the capacity for self-repair. (N10)*

*I don't want my mom to help me with bathing anymore; I want to bathe independently. I believe I am capable of doing it on my own. (N1)*

#### Sub-theme 4: cues to action

Most participants perceived clear benefits from seeking help, which encouraged them to visit the hospital more proactively. First, China's health insurance system significantly reduced their financial burden. Second, they believed that ongoing advances in medical technology gave them confidence in the effectiveness of care. Finally, family members often encouraged them to seek professional medical help, further supporting their decision to act.

*The government's policies are pretty good now and have greatly reduced the pressure. We feel well supported, so my mood has improved a lot. My surgery cost more than 110,000 yuan, but after insurance reimbursement, I only paid about 70,000. (N3)*

*I didn't want to go through so much suffering, but my son insisted I come for treatment. He told me not to worry. (N5)*

*Even when I cough lightly indoors at times, my family will grow quite worried. They have always supported me in seeking treatment. (N11)*

*If the doctor says this treatment plan isn't feasible, can I opt for an alternative one? There's also proton therapy available now, and I can apply for it. (N6)*

### Theme 2: barriers

#### Sub-theme 5: perceived internal barriers

##### Lack of health awareness

Lung cancer symptoms are often masked by comorbid conditions that exhibit similar symptoms, making it difficult for patients to distinguish between preexisting manifestations and emerging pathological signs (Tod and Joanne, 2010; Corner and Brindle, 2011). Consequently, recognizing symptoms and making an accurate diagnosis can be particularly challenging (Carter-Harris et al., 2015; Dobson et al., 2018). Patients with lung cancer may mistakenly attribute their symptoms to causes unrelated to lung cancer (Al Achkar et al., 2021). For instance, many participants attributed symptoms such as coughing to less serious conditions like choking on water, overwork, or a common cold. Some participants also purchased medicines. In addition, when lung cancer patients experienced symptoms, they often waited to see if the symptoms improved or tried managing them on their own (Birt et al., 2014; Walton et al., 2013; Sharf et al., 2005).

*Later, I thought I had choked on a sip of water when I coughed. After choking, my throat became hoarse, and my voice became very soft. I thought it was just choking and that it would get better after a while. (N1)*

*At first, I just had a bit of a cough. But I thought it was a cold, and my husband believed it was pharyngitis caused by a cold. (N7)*

*“I went to a small local clinic right away and bought some herbal medicine from the doctor to drink. (N13)*

*I don't usually cough, and my colds rarely involve fever. When I have a runny nose and sneezing, I just take some medicine. (N7)*

##### Conflict of personal responsibilities

In making decisions about seeking medical care, participants often struggled when work responsibilities conflicted with the need to see a doctor. Additionally, when they were personally responsible for caring for family members, they worried that seeking medical help would leave their loved ones without support.

*I was still working when I had symptoms. I started feeling unwell in the morning, and by 3 p.m. I had mostly finished my work. I could barely walk, and only then did I go to the hospital. (N4)*

*I caught a cold while taking care of my grandson there, and I didn't pay much attention to it. I thought I still had to look after my grandson, so I didn't go for a timely check-up. (N12)*

##### Psychological acceptance barriers

When confronted with this illness, patients often found it difficult to come to terms with their physical condition and the challenges they might face in the future. Additionally, some participants were in the stage of disease denial, attributing their illness to events from childhood.

*I just couldn't believe it. I don't smoke or drink at all. I hardly ever eat spicy or irritating foods, and I never eat barbecue from outside. I have no idea why I got this disease. In the end, doesn't this illness just lead to death? It leaves you with nothing. (N7)*

*I suspect my childhood role as a labor committee member, which involved extensive cleaning duties, might have contributed to this condition. (N8)*

##### Difficulties enduring treatment side effects

Participants reported experiencing multiple treatment-related side effects, including alterations in taste and smell perception, nausea, diarrhea, and appetite loss. The physical discomfort caused by these side effects often exacerbated patients' anxiety and fear. In cases of severe adverse reactions, some patients developed an aversion to treatment, which reduced their motivation to seek further medical care.

*Now, taking this clinical trial drug causes vomiting and diarrhea, and it's very uncomfortable. Everything I eat just passes right through, and my body is lacking nutrition. (N7)*

##### Significant economic pressure

The high cost of cancer treatment poses a significant burden, making it difficult for patients with limited financial resources to afford the necessary care. Some patients are unable to cover the high medical expenses and are consequently forced to forgo treatment.

*The financial burden of medical treatment for this disease poses significant challenges for average families. Given the chronic nature of the condition, while one to two years of treatment might be manageable, the escalating costs over an extended period become increasingly unsustainable for most households. (N9)*

*I've quit my previous job because of chemotherapy, and now I have no source of income. (N10)*

#### Sub-theme 6: perceived external barriers

##### Misdiagnosis

Some participants were not diagnosed with lung cancer during their first medical visit. Previous misdiagnoses and distrust in the healthcare system also hindered their subsequent help-seeking behavior (Saab et al., 2021b; Scott and Walter, 2010). From a disease perspective, the atypical nature of early lung cancer symptoms can lead to misdiagnosis (Wu et al., 2016). Both chest X-rays and chest CT scans may miss lung tumors (Del Ciello et al., 2017).

*My initial symptom was back pain, and my cough kept getting worse. I had tests done at the county hospital, and the doctor told me I had lung nodules but said everyone has them and that I just needed to quit smoking. I did quit, but I still didn't feel well. That doctor should have referred me to a better-equipped hospital. (N4)*

*Before I was diagnosed, I had already seen a doctor. When I had the cough, I visited a respiratory specialist, but he didn't order a CT scan. (N6)*

##### Long distance to medical facilities

Some participants lived in rural areas and reported that the long distance to healthcare facilities made seeking care difficult. The inconvenience of travel could discourage help-seeking, and the lengthy journey often placed emotional and practical burdens on both patients and their families.

*We came from Enshi, which is even farther away. The trip to the hospital takes a long time, so my son had to find a place to stay nearby. It's not easy for me to come here alone—getting medical care here is quite troublesome. But this treatment can only be done at this hospital. (N9)*

*My home is very far from this hospital, so treatment isn't convenient. Sometimes when I'm admitted, I have to bring a lot of things with me, which feels really burdensome. I also have to ask my family to take time off to care for me. (N12)*

## Discussion

Based on the HBM, this study investigated the facilitators and barriers to symptom-related help-seeking behavior among lung cancer patients in China, thereby expanding our understanding of how these patients respond to symptoms. Perceived threats, perceived benefits, self-efficacy, and cues to action all facilitated help-seeking among lung cancer patients. Barriers included perceived internal barriers and perceived external barriers.

According to the HBM, perceived susceptibility influences healthcare decision-making through the mediating effects of somatic perception and behavioral intention, ultimately affecting healthcare-seeking decisions (Luo et al., 2025). Somatic perception is sensitivity to bodily sensations and bodily activities caused by physiological changes (Luo et al., 2025). Consistent with previous studies conducted in Australia and Denmark, symptoms such as hemoptysis prompted patients to seek immediate medical care (Haastrup et al., 2020; Crane et al., 2016). Similarly, persistent coughing that showed no sign of improvement was more likely to trigger help-seeking than short-term coughing, a finding aligned with a prior study among patients in India (Shankar et al., 2016). This study is the first to propose “symptom interference with daily life” as a key criterion prompting lung cancer patients to initiate help-seeking.

This study also found that some lung cancer patients strengthened their help-seeking behavior after recognizing its benefits (Williams et al., 2022). First, by actively seeking help, patients gained a clearer understanding of their condition, which alleviated the confusion and psychological distress previously caused by uncertainty about their illness. Moreover, in China, middle-aged and younger adults are often the primary support for their families and carry significant family and social responsibilities alongside their treatment. Therefore, facilitating their return to work is crucial, not only to improve their psychological wellbeing and quality of life but also to reduce the financial burden on their families (Beesley et al., 2017; Pearce et al., 2019). For these patients, actively seeking help and receiving treatment represents not only an improvement in personal health but also the preservation of their family's livelihood.

The construct of self-efficacy involves an optimistic self-belief that one can manage novel or difficult tasks and handle life's adversities (Bandura, 1977). High self-efficacy is associated with increased likelihood to seek help, persistence in treatment, and empowerment to overcome barriers such as stigma and fear (Carlin et al., 2025; Gulliver et al., 2010). High self-efficacy is also linked to stronger self-management, which empowers individuals to support their own mental health by building the skills and confidence needed to actively recognize and manage their health challenges (Lean et al., 2019). Individuals with greater emotional regulation capacity tend to assess their symptoms calmly during health threats and seek medical care sooner (Aldao and Nolen-Hoeksema, 2012). This suggests that interventions aimed at improving symptom-related help-seeking behavior may be more effective if they also enhance self-efficacy (Amin et al., 2025).

Support provided by families, friends and healthcare systems collectively creates a social atmosphere advocating for seeking medical care (Jaiswal et al., 2018). This mechanism has been validated across cultural contexts: for example, research on Chinese populations shows that family and social support can effectively improve help-seeking behavior among lung cancer patients (Jiang et al., 2023). This may be attributed to the traditional Chinese concept of ‘family', where individuals are regarded as part of the family unit, rather than as entirely independent entities. Adequate social support facilitates early healthcare decisions and enhances positive coping strategies (Saldaña-Téllez et al., 2024); conversely, a survey of American populations found that reduced social support levels significantly increases difficulties in seeking medical help (Tambling et al., 2023). Therefore, communities can strengthen patients' social support networks in terms of emotional, informational, and practical care by organizing support groups for patients, thereby enhancing the quality and effectiveness of support.

Our study found that a lack of health awareness among participants hindered their help-seeking behavior. First, most participants attributed initial cancer symptoms to less serious conditions, such as the common cold or fever. Similarly, Australian lung cancer patients' vague understanding of symptoms has also been linked to delayed care-seeking (Malalasekera et al., 2021). Second, our research revealed that when experiencing physical discomfort, participants tended to habitually ignore symptoms or self-medicate rather than seek professional medical advice. This reflects the dual influence of limited health literacy and illness-related stigma in the general population (Aukst Margetic et al., 2020). The “watch-and-wait” approach is often perceived not merely as delayed help-seeking but as a frugal or pragmatic coping strategy. Communities can strengthen residents' awareness of lung cancer symptoms through health education activities and focus on conveying the positive message that “early diagnosis enables treatment and early treatment leads to cure.”

Our study indicates that patients' own role conflicts, psychological acceptance barriers, and inability to tolerate treatment side effects can impede their help-seeking behaviors. Due to competing priorities (such as caregiving responsibilities, work, and other life events), participants lacked sufficient time to seek medical help (Andersen et al., 2010; Burgess et al., 2006). This is associated with traditional Chinese culture. In China, “filial piety” and “family responsibility” are often endowed with high moral significance, and patients tend to prioritize family needs over their own health (Yu, 2024). When facing health issues, they are inclined to prioritize handling family affairs and regard medical consultation, examinations, or treatment as secondary matters. Cancer treatment not only causes physical suffering but also induces persistent psychological distress (Brown et al., 2023). Cancer patients often have complications, including mental health problems such as depression or anxiety (Sarfati et al., 2016; Jabaaij et al., 2012; Berry et al., 2014; Mausbach and Irwin, 2017; Mausbach et al., 2020). Negative perceptions of cancer outcomes and fear of consultation are factors contributing to delayed medical care among patients (Shahid et al., 2009; Forbes et al., 2013). Therefore, medical professionals should monitor patients' mental health, foster positive attitudes via counseling and health education, and highlight the value of early diagnosis and treatment. In addition, financial pressure remains a barrier to seeking medical assistance. Findings from a national survey in Portugal showed that 44.26% of delayed help-seeking behaviors among lung cancer patients were caused by insufficient economic capacity (Barata et al., 2021). Meanwhile, in the United States and Australia, high treatment and care costs are obstacles to lung cancer diagnosis (Cassim et al., 2019).

Perceived external barriers can impede their help-seeking behaviors. A study has shown that delays in seeking medical care mainly occur during the primary care interval, and the inaccessibility of secondary care is the primary cause of such delays (Vidaver et al., 2016). A study conducted in Australia also observed delays in medical care at the health system level, including delays in the referral process to specialists (Rankin et al., 2017). Therefore, it is crucial to further standardize the first-visit responsibility system and referral mechanisms, while attaching importance to the training and participation of general practitioners. In China, high-quality medical resources are mainly concentrated in provincial capitals and large cities, whereas medical resources in small cities and rural areas remain insufficient (Chai et al., 2020; Wu et al., 2022). In this regard, improving primary healthcare quality and building an effective early screening, diagnosis, and treatment network is critical. In recent years, China has implemented family doctor contracts and hierarchical diagnosis systems to boost primary medical service capacity and optimize medical resource allocation (Li et al., 2023; Gu et al., 2024; Xu et al., 2022). Communities need to strengthen collaboration with higher-level hospitals, open up a green channel for “community-based initial screening—rapid referral—diagnosis feedback” and shorten the time from the onset of symptoms to specialized diagnosis and treatment.

### Strengths

First, this study is among the first to apply the HBM to explore the facilitators and barriers of symptom help-seeking behaviors in lung cancer patients. It provides new insights into the roles of perceived disease threats, perceived benefits, self-efficacy, cues to action, and perceived barriers in these patients' symptom help-seeking behaviors. Second, this study adopted an HBM-guided thematic analysis method, enabling a deeper and more systematic exploration of the facilitators and barriers of help-seeking behaviors within a structured framework. Finally, the participants in this study showed diversity in age, place of residence, and educational level, which enhanced the reliability of the research findings.

### Limitations

First, all participants were recruited from a large Grade A tertiary hospital in Wuhan. This hospital concentrates high-quality medical resources and mainly admits lung cancer patients with severe conditions or those who have undergone multiple referrals. Thus, the sample cannot represent patients with early diagnosis or those who first seek treatment at primary medical institutions. Future studies that include patients from multiple hospitals or with different cultural backgrounds will help improve the representativeness of research results. In addition, as this qualitative study is based on interview data, participants' subjective factors (such as concerns about privacy or personal values) may lead to discrepancies between their described experiences and actual ones. However, this study effectively improved research reliability and reduced the impact of such biases by collecting extensive and rich data from participants.

### Conclusion

By interviewing 14 lung cancer patients, this study explored the facilitators and barriers of symptom help-seeking behaviors among lung cancer patients. The results indicate that lung cancer patients need to pay attention to their symptoms, enhance health awareness, improve willingness to seek medical care, and cope with the disease with a positive mindset. Relevant departments should continuously improve patients' medical experience, increase the accuracy of disease diagnosis, and strive to solve practical difficulties during patients' medical treatment to achieve better health outcomes. Meanwhile, society and family members need to provide patients with comprehensive support and back their medical decisions.

## Data Availability

The raw data supporting the conclusions of this article will be made available by the authors, without undue reservation.
