# Supplementary material for: Facilitators and barriers to help-seeking behavior for symptoms in patients with lung cancer in China—a qualitative study
Source: Front Psychol. 2026 Jan 12;16:1718034. doi: 10.3389/fpsyg.2025.1718034 (PMC12833010; doi:10.3389/fpsyg.2025.1718034)
Supplement: Supplementary file 1 [file Table_1.DOCX]

Table A1 Open Coding

| Categories | Raw data |
| --- | --- |
| Uncertainty about the illness | Coughing usually gets better in about two weeks, but I’d been coughing for three or four weeks. I felt really lost, so I went to the hospital for a check - up. |
| Fear of sudden acute symptoms | I suddenly had a severe muscle spasm at home and passed out from the pain. It really scared my husband, so we went straight to the emergency department at Zunyi Medical College for tests, including blood work and an MRI. |
| Interference with daily life | After falling asleep, my leg pain eased, but I still had sharp pain in my chest and back. It’s really affecting my daily life. |
| Alleviation of stress | Because we didn’t know what was causing my cough, my family and I felt it would be reassuring to get it checked. After the examination, my stress really eased. |
| Restoration of physical function | Since I got sick, I clearly noticed my body wasn’t as strong as before. But after getting help and starting treatment, my stamina improved. |
| Ability to earn an income | Before treatment, my hands weren’t as nimble. Now I can make handmade insoles at home and earn some money. |
| Strong emotion regulation skills | I usually maintain a calm attitude and don't burden myself with unnecessary thoughts. After coming to the hospital, I simply follow the doctor's guidance for treatment. |
| Confidence in rehabilitation capabilities | I am certain I can recover, and I am also willing to cooperate with treatment and exercise. |
| Health insurance policy support | The current national policies are highly favorable, significantly easing our financial burden. They have shown great consideration for us, which has greatly lifted our spirits. My surgery cost over 110,000 yuan, but after reimbursement, I only paid a little over 70,000 yuan. |
| Medical technical support | Given the significant advancements in medical treatments, I am initially taking first-generation drugs. Should resistance develop, I have the flexibility to switch to second- or third-generation drugs as needed. |
| Health information support | We are the kind of patients who are eager to learn, always searching for information online. We look into which drugs or treatments are available, or what surgeries could potentially cure us. |
| Family support for medical visits | I expressed my reluctance to endure such suffering, but my son insisted I seek treatment. He reassured me not to worry and to take it one step at a time, as long as the doctors could help. |
| Friend support for medical visits | At that time, I asked him, “it seems like I have cancer,” and a friend advised me to find a hospital for a check-up as soon as possible. Since I am illiterate, I didn’t understand what he meant. I have his WeChat, and he sent the tests I needed to my WeChat, so I took my phone to the hospital for the check-up. |
| Mutual support among patients | I communicate a lot with fellow patients to see what their treatment plans are like, and it feels good. At least it gives me some comfort. |
| Lack of health awareness | Later, I thought I had choked on a sip of water when I coughed. After choking, my throat became hoarse, and my voice became very soft. I thought it was just choking and that it would get better after a while. |
| Conflict of personal responsibilities | I was still working when I had symptoms. I started feeling unwell in the morning, and by 3 p.m. I had mostly finished my work. I could barely walk, and only then did I go to the hospital. |
| Psychological acceptance barriers | I just couldn’t believe it. I don’t smoke or drink at all. I hardly ever eat spicy or irritating foods, and I never eat barbecue from outside. I have no idea why I got this disease. In the end, doesn’t this illness just lead to death? It leaves you with nothing. |
| Difficulties enduring treatment side effects | Now, taking this clinical trial drug causes vomiting and diarrhea, and it’s very uncomfortable. Everything I eat just passes right through, and my body is lacking nutrition. |
| Significant economic pressure | I’ve quit my previous job because of chemotherapy, and now I have no source of income. |
| Misdiagnose | Before I was diagnosed, I had already seen a doctor. When I had the cough, I visited a respiratory specialist, but he didn’t order a CT scan. |
| Long distance to medical facilities | We came from Enshi, which is even farther away. The trip to the hospital takes a long time, so my son had to find a place to stay nearby. It’s not easy for me to come here alone — getting medical care here is quite troublesome. But this treatment can only be done at this hospital. |

TableA2 Axial Coding

| Sub-themes | Categories |
| --- | --- |
| Perceived threats | Uncertainty about the illness  Fear of sudden acute symptoms  Interference with daily life |
| Perceived benefits | Alleviation of stress  Restoration of physical function  Ability to earn an income |
| Self-efficacy | Strong emotion regulation skills  Confidence in rehabilitation capabilities |
| Cues to action | Health insurance policy support  Medical technical support  Health information support  Family support for medical visits  Friend support for medical visits  Mutual support among patients |
| Perceived internal barriers | Lack of health awareness  Conflict of personal responsibilities  Psychological acceptance barriers  Difficulties enduring treatment side effects  Significant economic pressure |
| Perceived external barriers | Misdiagnosis  Long distance to medical facilities |

Table3A Selective Coding

| Sub-themes | Themes | Main concept |
| --- | --- | --- |
| Perceived threats  Perceived benefits  Self-efficacy  Cues to action | Facilitators | Facilitators of help-seeking behavior for symptoms in lung cancer patients |
| Perceived internal barriers Perceived external barriers | Barriers | Barriers to help-seeking behavior for symptoms in lung cancer patients |
